# Spatio-temporal distributions and determinants of diarrhea among under-five children in Ethiopia

**DOI:** 10.3389/fpubh.2024.1369872

**Published:** 2024-05-20

**Authors:** Meskerem Tiku, Melkamu A. Zeru, Denekew Bitew Belay

**Affiliations:** ^1^Department of Statistics, Injibara University, Injibara, Ethiopia; ^2^Department of Statistics, Bahir Dar University, Bahir Dar, Ethiopia

**Keywords:** childhood, diarrhea, spatio-temporal, zones, Ethiopia

## Abstract

**Objective:**

The purpose of this study was to evaluate the spatio-temporal pattern of Ethiopia’s childhood diarrheal disease and identify its contributing factors.

**Methods:**

We conducted analyses on secondary data from four Ethiopian Demographic and Health Surveys conducted between 2000 and 2016. Moran’s I was used to determine spatial dependence and spatial models were used to evaluate variables associated with diarrhea in under-five children at the zonal level.

**Results:**

Childhood diarrhea showed spatial clustering in Ethiopia (Moran’s I; *p* < 0.05). The spatial regression model revealed significant factors at the zonal level: children born at home ( $e^{\theta }$ = 1.355, 95% CI: 1.052–1.544, *p* < 0.001), low birth weight ( $e^{\theta }$ = 1.18, 95% CI: 1.017–1.691, *p* < 0.05), and unimproved source of water ( $e^{\theta }$ = 0.8568, 95% CI: 0.671–1.086, *p* < 0.01).

**Conclusion:**

The prevalence of diarrhea among under-five children varied over time by zone, with the Assosa, Hundene, and Dire Diwa zones having the highest rates. Home births and low birth weight contributed to the prevalence of childhood diarrhea. In high-risk zones of Ethiopia, reducing childhood diarrhea requires integrated child health interventions and raising awareness about the potential hazards associated with unimproved water sources.

## Introduction

Diarrhea is the second leading cause of death and morbidity in children under five years worldwide and is primarily caused by contaminated food and water sources. The World Health Organization (WHO) defines diarrheal disease as the passage of three or more loose or liquid stools per day, or more frequent passage than is normal for the individual (1). In countries with lower incomes, it is more common and widespread due to its infectious nature (1, 2). In the world, more than 1.7 billion cases of childhood diarrheal disease occur every year (1). As the WHO reported in 2019, diarrheal disease is the second leading cause of death in children under five years, next to pneumonia, and was responsible for the deaths of 370,000 children (3). According to United Nations International Children’s Emergency Fund (UNICEF) report in 2021, 9.1% of all under-five children deaths in the world are caused by diarrhea diseases (2). From a global perspective, the spatial pattern of diarrhea-related mortality was clustered worldwide during the study period from 2000 to 2017. Furthermore, the findings indicated that the world’s highest incidence of diarrhea-related mortality was found in Asian and African nations (4).

In Africa, diarrheal diseases are the leading cause of death in children under five, accounting for an estimated 30 million cases of severe diarrhea and 330,000 deaths in 2015 (5). The highest mortality rate is in sub-Saharan Africa, where rates typically range between 50 and 150 per 100,000 population (6) depending on various factors such as mother’s age, wealth index, mother’s education, mother’s occupation, the age of the children, time of initiation of breastfeeding, and time to fetch water were significantly associated with diarrhea (7).

In Ethiopia, diarrhea was the major contributor to the deaths of children under the age of five years. The Ethiopian Demography and Health Surveys (EDHS) in 2000, 2005, 2011, and 2016 indicate that childhood diarrheal disease experienced by 24.72, 18.04, 16.1, and 11.79%, respectively, of children at some time in the two weeks before the survey (8–11).

In Ethiopia, childhood diarrhea remains public health problem and had a spatial variation across the regions. Although there was a decline in diarrhea rates nationally from 2000 to 2011, morbidity remained high in regions such as Gambella, Benshangul-Gumuz, and Somali (12). Childhood diarrheal disease is more common among children under five years of age, particularly in the Southern Nation and Nationality People (SNNP), Gambella, Oromia, and Benshangul-Gumuz regions (13). In a research setting, analyzing disease trends over time and space provides context that can be associated with potential risk factors. Scanning statistics have been extensively used in studying the spatial, temporal, and spatiotemporal variation of infectious diseases such as diarrhea (14).

Morbidity due to diarrhea has been found to be influenced by socio-demographic factors such as sex, age, family size, place of residence, wealth index, parental education, mothers’ age, and child’s age (7, 15–18), child-caring factors include immunization status, birth place, birth order, the child’s birth weight, and the child’s nutritional status (17, 19, 20). Housing and sanitation factors include drinking water sources, waste disposal, and toilet facilities (15, 21, 22), and meteorological factors include precipitation and temperature across the globe, including Ethiopia (23–25).

It is important to explain how diarrheal diseases occur in temporal and spatial variations to enable health professionals at different levels to make informed decisions and improve disease surveillance. Nevertheless, there are not many studies in Ethiopia that examined zonal diversity in diarrhea from a geographical and temporal perspective. Few studies that are now available used a variety of data sources for a spatio-temporal study of the spread of diarrhea, but they were only able to look at local rather than national data. Therefore, the aim of this study was to examine the variability of diarrhea in children in different geographical and temporal zones of Ethiopia. The study adds to the body of information and identifies risk zones to enable the development of effective intervention techniques to reduce pediatric diarrhea in Ethiopia.

## Methods

### Description of study area and study setting

**Figure 1:**
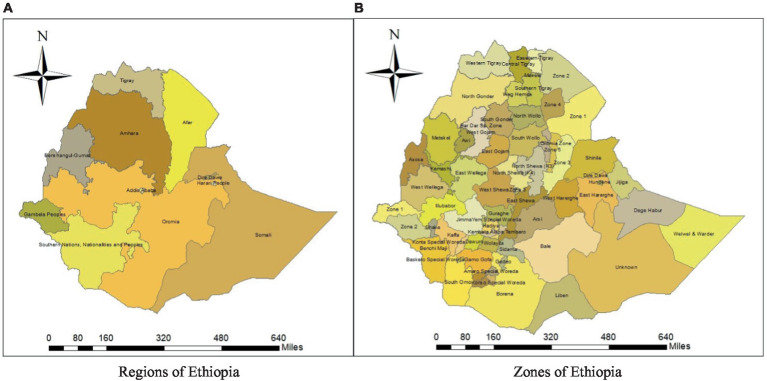
The map of Ethiopia: **(A)** 11 regions and **(B)** 72 zones.

This study was conducted in Ethiopia, which is situated in eastern Africa. It is a landlocked country in the Horn of Africa, located between 3° and 15° north latitude and 33° and 48° east longitude (EDHS, 2000). The country has 11 regional states and two administrative cities, Addis Ababa and Dire Diwa (Figure 1).

### Data collection and sampling procedure

The data was obtained from the Ethiopian Demographic and Health Survey (EDHS). The DHS results are available for many nations, particularly those with low incomes. The DHS data is readily accessible and publically available at: https://dhsprogram.com. In order to offer the most accurate data on indicators related to maternal and child health, the EDHS data is generated from a series of nationally representative demographic and health surveys conducted in the country every five years. This study took into account four EDHSs that were completed in 2000 along with those in 2005, 2011, and 2016, allowing for a summary of the results for the entire nation. A stratified two-stage cluster sampling procedure was used to select the nationally representative sample in all the four surveys. In the first stage, clusters or enumeration areas (EAs) were selected with probability proportional to population size. A total of 540, 540, 624 and 645 EAs (clusters) were selected in 2000, 2005, 2011, and 2016, respectively. The second stage involved the stratified sampling of households in each selected cluster. The study was conducted on a sample of 64,449 children: 18,060 from 2016; 17,817 from 2011; 14,500 from 2005; and 14,072 from the 2000 EDHS, respectively.

The dependent variable for the $i^{\mathrm{th}}$ childhood diarrheal status was represented by a random variable with two possible values coded as 1 and 0. The occurrence of diarrhea was classified as “yes” or “no” by asking mothers whether their children had an incidence of diarrhea in the last two weeks before the survey. It needs some clarification, such the number of under-five children with diarrheal disease within two weeks before the survey is undertaken. Therefore, the response variable of the $i^{\mathrm{th}}$ childhood diarrhea occurrence in space (zone) was measured as a count variable.

Childhood diarrheal status is expected to be influenced by several factors. Therefore, the predictor variables that were analyzed in this study as possible determinants of diarrhea among under-five children were grouped into socio-economic and socio-demographic factors, child-caring practice, geographical covariate, and housing and sanitation related factors (Table 1).

**Table 1:** The descriptions of variables included in the model.

Covariates	Description
Children have diarrhea	The number of children who have diarrhea
Male children	The number of male children
Infants	The number of children aged 0–12 months
Young age mothers	The number of mothers aged 15–24 years
Above two children in household	The number of households with >2 children
Single marital status	The number of single marital status family
Rural residence	The number of rural places of residence
An illiterate mother	The number of illiterate mothers
An illiterate father	The number of illiterate fathers
Mothers studying status	The number of mothers with working status
Media exposure	The number of family with have no media exposure
Wealth index	The number of households with a high poverty rate
Unimproved water source	The number of households with unimproved drinking water source
Improper toilet facility	The number of households with improper toilet facility
Unsafe stool disposal	The number of households with improper stool disposal
Children with no breastfeeding	The number of no breastfeeding of children
Children with no vaccinated	The number of unvaccinated of children
Malnourished children	The number of children having at least one from stunting, wasting, and underweight
Children with multiple birth type	The number of children with multiple birth type
< 2 years birth interval	The number of <2 years preceding birth interval
Low birth weight	The number of children with low birth weight
Home delivery	The number of home deliveries
Above second birth order	The number of children above second birth order
Average Precipitation	The average precipitation measured
Average Aridity	The ratio of annual precipitation to potential evapotranspiration
Average Max temperature	The average annual maximum temperature
Average Min temperature	The average annual minimum temperature
Average Potential Evaporation	The average PET
Average Wet days (WetD)	The average number of days receiving rainfall

### Data analysis

Global spatial autocorrelation is a measure of the overall clustering of the data, providing a correlation value to summarize the entire study area using the Moran I statistic. The Moran’s I values, which is found in the interval of (−1,0) , indicate the disease dispersed, whereas Moran’s I values found in (0,1) indicate disease clustered and disease distributed randomly if Moran’s I value is zero. Moran’s I statistic of spatial autocorrelation has the form described in equation given below (26).


$$I=\frac{N}{\sum_{i}^{N}\sum_{j}^{N}w_{ij}}\frac{\sum_{I}^{N}\sum_{J}^{N}w_{ij}\left(y_{i}-\bar{y}\right)\left(y_{j}-\bar{y}\right)}{\sum_{i}^{N}{\left(y_{i}-\bar{y}\right)}^{2}}$$

In this study, the spatial interpolation technique was used to predict unsampled from sampled measurements and ordinary Kriging spatial interpolation method was used for predictions and to generate smooth surfaces of the variables of interest (27).

The Scan statistics are used to detect and evaluate clusters of cases in either a purely temporal, purely spatial or space–time setting. This is done by incrementally scanning a window across time and/or space, noting the number of observed and expected observations within the window at each location (28). It is preferred among software programs capable of space–time disease surveillance analysis, also found to be the best-equipped package for use in surveillance system (29). In this study, the number of diarrheal cases, the number of under-five children population, and the coordinates of the study areas were used as input variables for the discrete Poisson model, assuming that the cases in each district have a Poisson distribution with a known population of under-five children that are at risk for diarrhea.

To detect and analyze the spatial clusters of childhood diarrhea, a purely spatial analysis was carried out using spatial scan statistics without taking time into account. This spatial statistical analysis method imposes a circular window on the map. The recommended choice is to express the upper limit as a percentage of the population at risk and use 50% as the value (28). Therefore, in this study, the maximum spatial cluster size of the population at risk is set at 50% as an upper limit, which allowed both small and large clusters to be detected and clusters that contained more than the maximum limit to be ignored.

Spatial regression models allow us to account for dependencies between observations, which often occur when collecting observations from points or regions in space (30, 31). Let y denote N×1 column vector of observations on the response variable, X denote N×k matrix of observations on the independent variables, and ε is N×1 vector of the error term.

Let W be a spatial weight matrix with a dimension of 72×72 , WX denote the interaction effects among the independent variables with the spatial components, and Wu denote the interaction effects among the error terms of different observations with the spatial components, and Wy represent the spatial lag vector. As regards the parameters of the model, ρ is the spatial dependence parameter, θ is the parameter of lagged independent variables, λ is the spatial correlation effect of errors (spatial autocorrelation).

The number of childhood diarrheal cases in zone i(i=1,2,..,72) , $\;y_{i}$ , were assumed to follow a Poisson distribution with mean $\lambda_{i}$ ,


$$y_{i}\sim \mathrm{poisson}\left(\mu_{i}\right)$$

**Figure 2:**
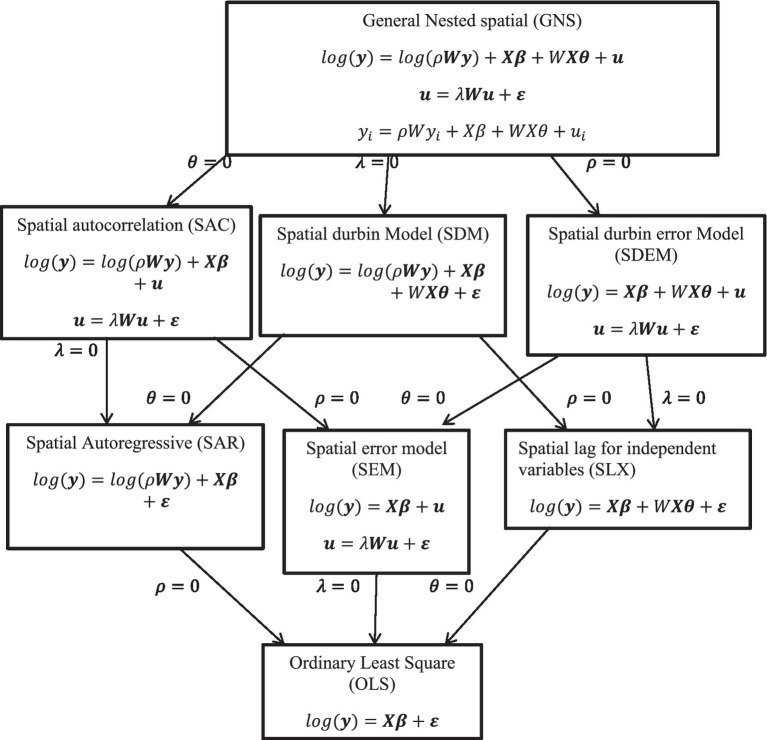
Diagrammatical presentation of spatial models.

Different spatial models were used with the addition of different covariates to determine the best and appropriate model for this study (Figure 2).

## Results

The prevalence of diarrhea among children under five years in Ethiopia was 24.72, 18.04, 16.10, and 11.79% in 2000, 2005, 2011, and 2016, respectively. Its was higher in rural areas (20.45, 16.27, 13.57 and 9.61%) over the four EDHS in 2000, 2005, 2011, and 2016, respectively, compared to urban areas. In a similar way, the prevalence of diarrheal case was higher in boys (12.26% in 2000, 9.37% in 2005, 8.38% in 2011 and 6.33% in 2016) compared to girl under-five children over those four-survey time periods. From this result, the prevalence of the childhood diarrhea disease decreased over time in the four EDHS phases (Table 2).

**Table 2:** The distribution of diarrheal cases in four EDHS data.

Independent variable	Categories	Frequency (%) of childhood diarrhea disease case			
		2000	2005	2011	2016
Place of residence	Rural	1757 (20.45)	1,370 (16.27)	1,305 (13.57)	962 (9.61)
	Urban	256 (2.98)	176 (2.09)	210 (2.18)	218 (2.18)
Mother’s education	Uneducated	1,660 (19.32)	1,187 (14.1)	1,051 (10.93)	708 (7.08)
	Educated	353 (4.1)	359 (4.26)	464 (4.82)	472 (4.72)
Father’s Education	Uneducated	1,316 (15.32)	901 (10.7)	755 (7.85)	512 (5.12)
	Educated	697 (8.11)	645 (7.66)	760 (7.9)	668 (6.68)
Source of water	Unimproved water	707 (8.23)	662 (7.86)	768 (7.98)	458 (4.58)
	Improved water	1,306 (15.2)	884 (10.5)	747 (7.76)	722 (7.22)
Toilet facility	Unimproved	1,676 (19.51)	1,419 (16.85)	1,321 (13.73)	991 (9.9)
	Improved	337 (3.92)	127 (1.51)	194 (2.02)	189 (1.89)
Mother’s age	Young aged	564 (6.57)	427 (5.07)	379 (3.94)	333 (3.33)
	Old aged	1,449 (16.87)	1,119 (13.29)	1,136 (11.8)	847 (8.46)
Stool disposal	Unsafe	1798 (20.93)	1,174 (13.94)	1,019 (10.59)	685 (6.85)
	Safe	215 (2.5)	372 (4.42)	496 (5.16)	495 (4.95)
Mother’s work	Not working	894 (10.41)	1,146 (13.61)	1,016 (10.56)	815 (8.15)
	Working	1,120 (13.04)	400 (4.75)	499 (5.19)	365 (3.65)
Breastfeeding status	Yes	1,551 (18.06)	1,199 (14.24)	378 (3.93)	373 (3.73)
	No	462 (5.38)	347 (4.12)	1,137 (11.82)	807 (8.07)
Marital status	Single	172 (2)	125 (1.48)	182 (1.89)	92 (0.92)
	Married	1841 (21.43)	1,421 (16.87)	1,333 (13.86)	1,088 (10.87)
Birth order	≤2 birth order	706 (8.22)	527 (6.26)	507 (5.27)	460 (4.6)
	>2 birth order	1,307 (15.22)	1,019 (12.1)	1,008 (10.48)	720 (7.19)
Child sex	Male	1,053 (12.26)	789 (9.37)	806 (8.38)	633 (6.33)
	Female	960 (11.18)	757 (8.99)	709 (7.37)	547 (5.47)
Child age	≤12 months	512 (5.96)	428 (5.08)	436 (4.53)	326 (3.26)
	>12 months	1,501 (17.47)	1,118 (13.28)	1,079 (11.22)	854 (8.53)
Birth type	Multiple	22 (0.26)	16 (0.19)	46 (0.48)	24 (0.24)
	Single	1991 (23.18)	1,530 (18.17)	1,469 (15.27)	1,156 (11.55)
Birth interval	≤24 months	452 (5.26)	354 (4.2)	332 (3.45)	305 (3.05)
	>24 months	1,561 (18.17)	1,192 (14.16)	1,183 (12.3)	875 (8.74)
Birth place	At home	1861 (21.66)	1,405 (16.69)	1,361 (14.15)	733 (7.33)
	At hospital	152 (1.77)	141 (1.67)	154 (1.6)	447 (4.47)
Birth weight	Low	722 (8.4)	480 (5.7)	519 (5.4)	403 (4.03)
	Normal	1,291 (15.03)	1,066 (12.66)	996 (10.35)	777 (7.77)
Vaccinated child	No	541 (6.3)	626 (7.43)	388 (4.03)	306 (3.06)
	Yes	1,470 (17.11)	920 (10.93)	1,127 (11.72)	874 (8.73)
Malnutrition	Yes	1,322 (15.39)	1,248 (14.82)	838 (8.71)	674 (6.74)
	No	691 (8.04)	298 (3.54)	677 (7.04)	542 (5.42)
Wealth status	Poor	1,101 (12.82)	874 (10.38)	744 (7.73)	584 (5.84)
	Rich	912 (10.62)	672 (7.98)	771 (8.02)	596 (5.96)
Number of children	≤2 children	1725 (20.08)	1,306 (15.51)	1,233 (12.82)	1,007 (10.06)
	>2 children	288 (3.35)	240 (2.85)	282 (2.93)	173 (1.73)
Media exposure	No	588 (6.85)	566 (6.72)	777 (8.08)	408 (4.08)
	Yes	1,425 (16.59)	980 (11.64)	738 (7.67)	772 (7.72)

**Figure 3:**
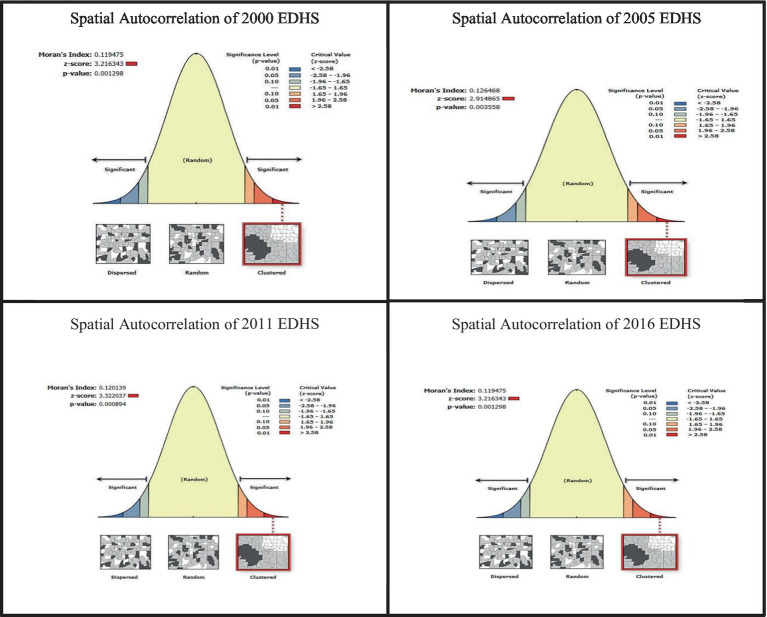
The spatial autocorrelation of childhood diarrheal cases.

All four Moran’s I *p*-values of childhood diarrhea were less than 0.05 for all the EDHS data; it implies that there exists a significant spatial autocorrelation in risk of childhood diarrhea disease between zones of the country (Figure 3). All Moran’s I index values were positive and indicate that the spatial distribution of childhood diarrhea disease was significantly clustered between zones of the country (p−value<0.01) .

**Figure 4:**
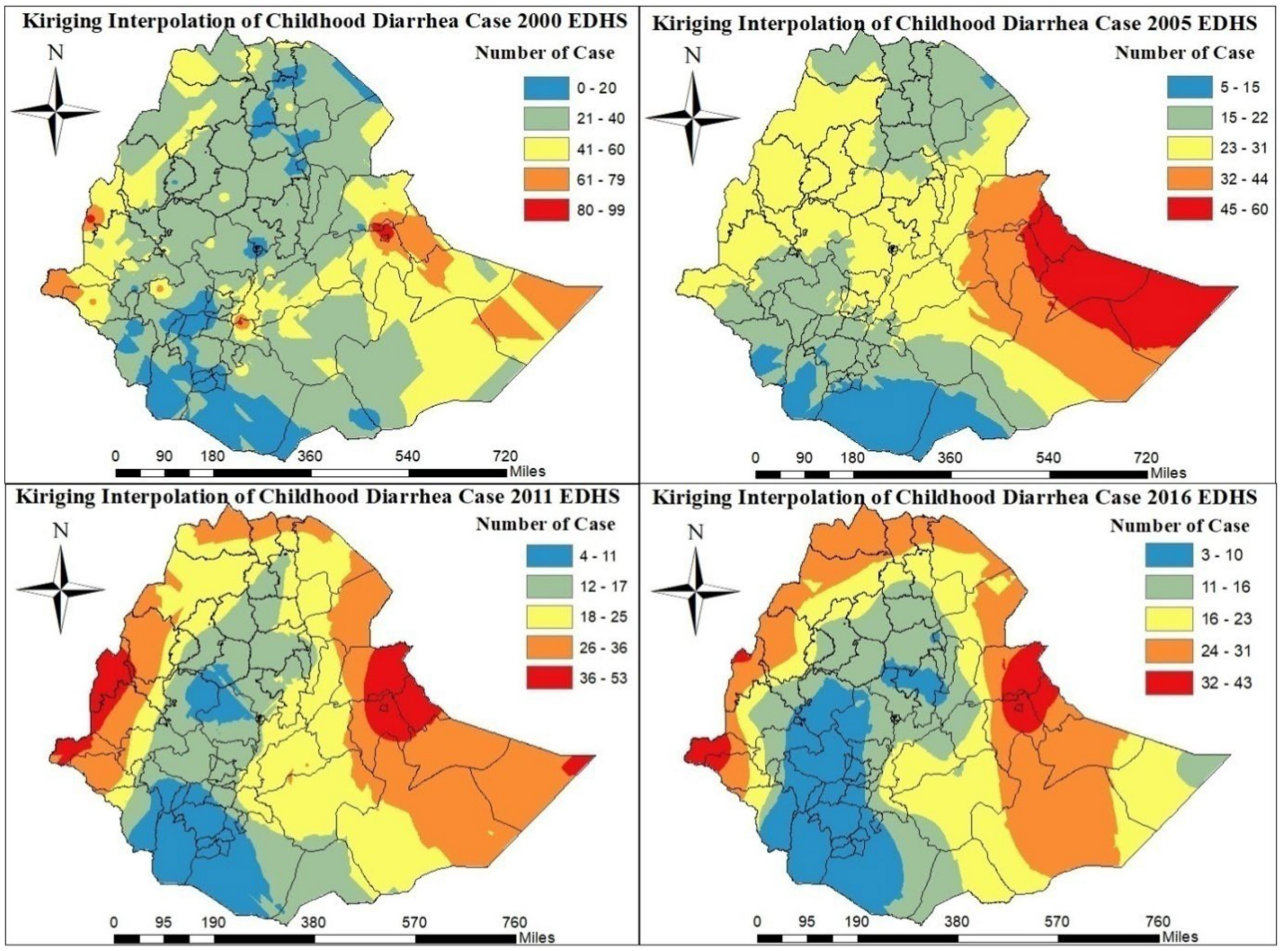
Kriging interpolation of childhood diarrheal from the 2000–2016 EDHS.

Kriging interpolation shows that the western and eastern areas of the country were heavily affected by childhood diarrhea in the 2000–2016 EDHS. In addition, in the 2011 and 2016 EDHSs, the northern part zones of the country were heavily affected by childhood diarrheal disease (Figure 4). Conversely, as shown in Figure 4, according to the EDHS 2000–2016, there are few cases of childhood diarrhea in most zones in the central and southern parts of the country.

**Figure 5:**
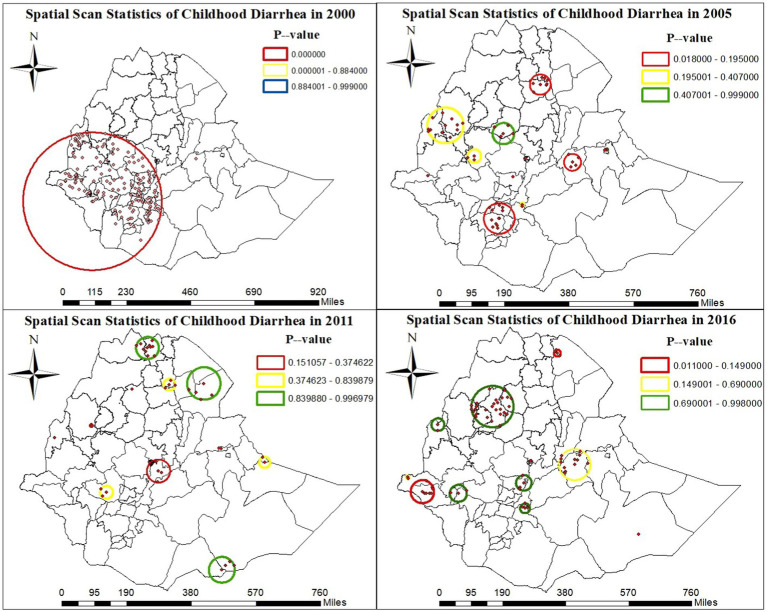
Spatial scan statistics of childhood diarrhea from the 2000–2016 EDHS.

A total of 186 significant clusters (164 in 2000 EDHS, 18 in 2005 EDHS, and 4 in 2016 EDHS) were identified. Of the overall significant clusters, 173 clusters were primary clusters: 164 in the 2000 EDHS survey and the remaining clusters were in the 2005 and 2016 EDHS. Thirteen clusters were secondary clusters in the 2005 EDHS survey. The primary and secondary spatial cluster windows in the 2000 EDHS were found in the west and south-west zones of the country. In the 2005 EDHS, these windows were found in West Hararge, around the Wolayita, Gedeo, Dawro, Sidama, and GamoGofa zones, and in the 2016 EDHS, they were found in Afar Zone 2 (Figure 5).

The model with the smallest Akaike Information Criterion (AIC) value was the best model. The spatial Durbin model was the best model for the 2000 EDHS data (AIC=468.1866) and the general nested spatial model was the best model for 2011 EDHS data (AIC=360.2615) (Table 3). In the same table, the spatial Durbin error model was the best for the 2005 and 2016 EDHS data (AIC = 310.1889 and AIC = 381.8517) respectively (Table 4).

**Table 3:** The estimated parameter ( θ ) of spatial regression models.

Covariates	2000 EDHS	2005 EDHS	2011 EDHS	2016 EDHS
	SDM	SDEM	GNS	SDEM
Intercept	−23.804	64.16850698	−12.3301	81.14401*
Average annual perception	0.388229	0.621400815**	0.08544	−0.24105
Average annual aridity	−1.31449	−2.37247091**	−0.48907	0.309124
Average Max Temp	2.596359	−4.815759821**	2.378274	−2.73426*
Average Min Temp	−4.09121	3.610674223**	−3.84971	0.036489
Average annual PET	4.893611*	0.997900695	3.015946	4.132153*
Average annual WetD	−2.30557*	1.964238905	−1.14045	−1.49412
Rural resident	0.222171	0.318009281**	−0.11845	0.099586
Illiterate mother	0.750718	0.20482172	0.399777*	−0.78582***
Illiterate fathers	0.70637**	−0.078987566	−0.27204*	0.344126*
Unimproved water	−0.03447	−0.28234542**	−0.19486**	−0.02803**
Unsafe toilet facility	0.785589	−0.325366185*	−0.32921*	0.04649
>2 child in household	0.528082	−0.48819163**	0.460418**	0.090874
<24 aged mothers	0.734182*	0.215760447*	−0.13669	−0.24357
Unsafe stool disposal	−1.35165	−0.6469349***	0.308216*	0.0303
Mothers who are workers	−0.00783	0.227462637*	0.106424*	0.246713
No breastfeed child	−0.01671	−0.254384517	−0.02265	0.460677**
Single marital status	−0.20561*	0.571689846*	0.60259**	−0.28084
>2 birth order	−0.72489	−0.216319328	0.05426	0.092005
Multiple birth type	1.904602	−0.163179307**	0.436498*	1.347798**
Male children	0.526696	0.167581181	−0.25285	−0.49859*
Infant	0.760907	0.253812624*	0.114946	−0.08373*
<2-year birth interval	−0.65495	0.534397174**	0.215775	0.064586
Home deliver	0.057493	0.382938222**	0.295565	0.243178**
Low birth weight	−0.02372	0.2072785	0.138724*	0.271174*
Unvaccinated children	−0.06801	0.248529924**	−0.51842**	−0.18538
Children with malnutrition	0.648074	0.256061368*	0.181526	−0.19448**
No media exposure	−0.21601	−0.35116232***	−0.45413*	0.065201
Poor and poorer family	0.296912	0.284017485***	0.220332**	0.050419
Rho ( ρ )	−0.3338*		−0.267367	
Lambda (𝜆)		−0.561*	−1.073363*	−0.417920
Adjusted R-square	0.9412	0.9886	0.9743	0.9434

“*” significance at 5%, “**” significance at 1%, “***” significance at 0.1%.

**Table 4:** The spatial regression models with AIC of all EDHS data.

Spatial models	AIC (Akaike Information Criterion) values			
	2000 EDHS	2005 EDHS	2011 EDHS	2016 EDHS
SAC	520.7712	441.104	441.8375	445.1616
SAR	529.9293	441.7932	442.6012	443.367
SDM	468.1866	355.6594	393.6559	391.4771
SDEM	470.8823	310.1889	371.7989	381.8517
SEM	517.4044	445.4591	439.655	443.4225
GNS	468.6132	321.3401	360.2615	383.5784
SLX	494.1551	358.1446	412.3824	389.5212

The adjusted R-squared values in the four EDHS phases showed how the prevalence of diarrhea in children was explained by the set of explanatory variables. All adjusted R-square values of the four EDHS data were above 90%, indicating that more than 90% of the covariates at different levels were good at explaining the cases of diarrhea in under-five children (Table 3).

From the results of Table 3, the geographical covariates such as maximum temperature, potential evaporation, and annual perception are significant factors for childhood diarrheal cases. In the 2005 EDHS data, zones with a higher average precipitation are positively associated with a higher mean number of diarrheal cases among children under-five ( $e^{\theta }=1.46,p-value<0.05$ ).

Some socio-demographic factors have a significant influence on the number of cases of diarrhea in children under-five (Table 3). In the 2005 EDHS data, zones with a higher number of children from rural zones are positively associated with a higher average number of diarrheal cases among children under five years ( $e^{\theta }=1.12,p-value<0.05$ ).

As shown in Table 3, some socio-economic factors such as illiterate mothers, illiterate fathers and working mothers are important contributing factors for diarrhea in children under-five. The zones with a higher number of illiterate mothers are positively associated with a higher mean number of diarrheal cases in children under-five in the 2011 EDHS data and negatively in the 2016 EDHS data ( $e^{\theta }=1.27\;and\;0.817,\mathrm{respectively},p-value<0.05$ ). In the 2011 EDHS data, zones with a higher number of illiterate fathers are negatively associated with a higher mean number of diarrheal cases among children under-five ( $e^{\theta }=0.844,p-value<0.05$ ).

In child-caring practice, some factors are significant for the number of childhood diarrheal cases as shown in Table 3. In the 2016 EDHS data, the zones with a higher number of low-birth-weight children are positively associated with a higher mean number of childhoods diarrheal cases $\;(e^{\theta }=1.18,p-value<0.05$ ). The zones with a higher number of children born at home are negatively associated in the 2005 EDHS data and positively associated in the 2016 EDHS data with a higher mean number of childhoods diarrheal cases $(e^{\theta }=0.74\;and\;1.355,\mathrm{respectively},p-value<0.01$ ).

Unimproved water supply, unimproved toilet facilities, and unsafe stool disposal are factors listed in Table 3 that are also significant in sanitation and hygiene practices for diarrheal disease in children under five years of age. Zones with a higher number of households with unimproved water supply are negatively associated with a higher mean number of diarrheal cases in under-five children in the 2011 and 2016 EDHS data ( $e^{\theta }=0.8236\;and\;0.8568,\mathrm{respectively},p-value<0.05$ ).

In the 2005 and 2011 EDHS data ( $e^{\theta }=0.722\;and\;0.7194,\mathrm{respectively},p-value<0.05$ ), the increase in the mean number of childhood diarrheal cases is negatively associated with the increasing number of households that have unimproved toilet facilities. The increasing the number of households with unsafe stool disposal is negatively associated in the 2005 EDHS and positively associated in the 2011 EDHS with the increasing mean incidence of childhood diarrhea $\left(e^{\theta }=0.523\;and\;1.361\mathrm{,respectively,}p-value<0.05\right)$ .

The spatial coefficient rho ( ρ ) was statistically significant ( p−value<0.05 ) in the 2000 EDHS data. This indicates that childhood diarrhea was significantly influenced by the adjacent zones. In the 2005–2011 EDHS data, areas with a higher number of unobserved variables (λ ) are negatively associated with a higher mean number of diarrheal cases in children under the age of five ( p−value<0.05 ) (Table 3).

## Discussion

Nationally, the number of children under-five exposed to diarrheal disease fell from 2,360 in 2000 to 1,180 in 2016. Although the general trend of reducing diarrheal disease in children under-five in Ethiopia over time is good, local trends show obvious heterogeneity. This study finding is similar with the study conducted in Ethiopia and India (12, 17, 32, 33) while the finding is different from the study conducted in Ghana (34). This could be because each county has different knowledge about the causes of diarrhea, the study environment is different, and there are differences in the study area.

The results of this study showed that childhood diarrheal diseases have a statistically significant and positive spatial autocorrelation in all zones of the country during the EDHS period 2000–2016. This study result is similar to the results of the study conducted in Ethiopia, India, Brazil, Rwanda, Nigeria, Ghana, and Tanzania (12, 13, 15, 16, 34–42) while this finding is different from the study conducted in Nepal (43). The possible reason for this difference could be the sample size, temperature, and lack of access to drinking water, especially in remote areas.

Furthermore, the results of this study showed that the Dire Diwa, Hundene, Jijiga, Dege Habour, and Welwel and Warder zones were highly affected by childhood diarrhea. This finding is also similar to the findings of the study conducted in Ethiopia (12) but different from the results of the study conducted in Ethiopia (13). This difference was probably due to Dire Diwa, Harari, and Somali regions lying in the low land part of the country. Conversely, zones in Addis Ababa, Jimma, Wolayita, Gamo Gofa, North Shewa, South Gondar, West Gojjam, and South Wollo zones areas with a low risk of childhood diarrhea, which is different from the results of the study conducted in Ethiopia (12, 13). The risks were lower in middle part of the country, which might be due to urbanization, improved drinking water sources, and high land areas of the country.

The scan statistics of spatial cluster showed that children inside the windows located in Gambela zone 1 and zone 2, Kamashi, Ilubabor, West Wellega, Kamashi, Shaka, Benchi Maji, Jimma, Hadiya, Wolayita, Assosa, West Harerge, Wolayita, Gedeo, Dawro, Sidama, Gamo Gofa, and Afar Zone 2 zones are more than once at the risk of diarrhea than children outside windows, according to 2000–2016 EDHS data. This study finding is similar with study findings that were reported from Ethiopia (12, 13).

In our study, some socio-demographic and socio-economic factors are significant for the childhood diarrhea. Based on the results of the spatial model, the illiteracy of both father and mother are directly proportional to the increase in the mean number of diarrhea of childhood diarrheal cases in children under the age of five. This result is in line with the previous studies conducted in Sub-Saharan Africa and Ethiopia (7, 17, 44) while this result is different from the previous studies conducted in East Africa and Nigeria (13, 15, 21, 45). This could be because the sample data taken from administrative zones of the country is different in different years and also due to the difference in study settings.

The study finding revealed that in spatial models, increasing number of illiterate fathers leads to an increase in the mean number of childhood diarrheal cases. This result is different from the result of the study conducted in India (32). This could be because the illiterate father does not have enough knowledge about the cause of diarrheal disease and its prevention.

In this study, some child-caring practices are significant for childhood diarrheal disease. This study results, based on a spatial model, showed that the increasing number of children who are twins and are born with low birth weight lead to an increase in the mean number of diarrheal cases among children under five years of age. Our study result is in line with the previous study results that were reported from Ethiopia and India (17, 18, 32). The possible reason might be due to mothers not caring for twin children properly and could be due to the fact that children with low birth weight have infant health problems or maternal health problems. Furthermore, children whose birth was at home increases the mean number of diarrheal cases, because of there is no safety at delivery time and initiation of breastfeeding and mothers did not get any advice from a nurse or a doctor about child care practices when they had birth at home. This finding agrees with the study conducted in Ethiopia (46).

The present study finding confirmed that children, who did not get breastfed, had higher mean number of diarrheal cases. The possible reason might be children who did not get breastfed miss out on the protective antibodies and immune factors present in breast milk. Lack of breastfeeding for children compromises their immune system, making them more vulnerable to pathogen invasion, which can lead to an increased risk of developing the diarrheal disease. Moreover, breast milk plays a crucial role in providing natural defenses against pathogens, promoting a healthier immune response, and reducing the likelihood of diarrhea in infants. This finding is similar to study results reported from Germany and Saudi Arabia (47, 48).

### Limitations of the study

Since this study used EDHS data from 2000 to 2016, the results could not accurately reflect the current state of affairs in Ethiopia. Additionally, other significant variables like respondents’ cultural, behavioral, and other health-related aspects were not included in the data set. We looked at a lot of potential causes of diarrhea in children in our study, but we might have missed other unrecognized or unidentified diverse contributors in the spatial dimensions.

## Conclusion

Although diarrhea has occasionally occurred in children under five years of age, its incidence is still high. Despite a falling pattern of childhood diarrhea at the national level, the prevalence remained high in the Assosa, Hundene, and Dire Diwa zones and displayed spatial and temporal variations throughout the country’s zones. Moreover, the eastern regions of the nation, including Jijiga, Dege Habour, Afar zones 1, Afar zone 3, Afar zone 4 and Hundene were hotspot areas of child diarrheal cases. Increases in the number of children due to multiple births, home births, children living in rural areas, low-birth-weight infants, unvaccinated children, households with unsafe stool disposal practices, malnourished children, both fathers and mother being illiterate were associated with an increase in the mean number of diarrheal cases among under-five children. Furthermore, use of unimproved water, unimproved toilet facilities, and unsafe stool disposal had significant effect for the high prevalence of diarrheal disease in under-five children. Thus, this study suggests that in order to prevent and control diarrheal, attention should be given for the above mentioned risk factors and appropriate intervention mechanisms should be applied. Moreover, the results of this study emphasizes public health education, vaccination program, provision of safe drinking water, and constant surveillance to find out the etiological agents causing diarrhea.

## Data availability statement

The data sets used for this study are available at the DHS program, in the DHS repository (https://www.dhsprogram.com/data/dataset/Ethiopia_Standard-DHS_2016.cfm?flag=0) for all four surveys.

## Ethics statement

Ethical approval for this research was obtained from ethical approval committee of postgraduate and research office, the College of Science, Bahir Dar University, Ethiopia. In the time of data collection there was no verbal consent from study participants because of the data was taken from secondary source from Ethiopian Demographic Health Survey data (EDHS from 2000 to 2016).

## Author contributions

MT: Conceptualization, Formal analysis, Methodology, Software, Writing – original draft, Writing – review & editing. MZ: Conceptualization, Formal analysis, Methodology, Software, Supervision, Writing – original draft, Writing – review & editing. DB: Formal analysis, Methodology, Software, Supervision, Writing – original draft, Writing – review & editing.
